# A comparison of specialist rehabilitation and care assistant support with specialist rehabilitation alone and usual care for people with Parkinson's living in the community: study protocol for a randomised controlled trial

**DOI:** 10.1186/1745-6215-12-250

**Published:** 2011-11-23

**Authors:** Heather Gage, Sharlene Ting, Peter Williams, Karen Bryan, Julie Kaye, Beverly Castleton, Patrick Trend, Derick Wade

**Affiliations:** 1 Dept of Economics, University of Surrey, Guildford, Surrey GU2 7XH, UK; 2 Dept of Mathematics, University of Surrey, Guildford, Surrey GU2 7XH, UK; 3 Division of Health and Social Care, University of Surrey, Guildford, Surrey GU2 7TE, UK; 4 NHS Surrey, Cedar Court, Guildford Road, Leatherhead, Surrey, KT22 9AE, UK; 5 Movement Disorders Service, Ashford St Peter's Hospital, Guildford Road, Chertsey, Surrey, KT16 9BN, UK; 6 Department of Neurology, Royal Surrey County Hospital, Egerton Road, Guildford, Surrey, GU2 7XX, UK; 7 Oxford Centre for Enablement, Windmill Road, Oxford, Oxfordshire, OX3 7LD, UK

**Keywords:** Parkinson's disease, Multidisciplinary rehabilitation, Domiciliary, Randomised controlled trial

## Abstract

**Background:**

Parkinson's Disease is a degenerative neurological condition that causes movement problems and other distressing symptoms. People with Parkinson's disease gradually lose their independence and strain is placed on family members. A multidisciplinary approach to rehabilitation for people with Parkinson's is recommended but has not been widely researched. Studies are needed that investigate cost-effective community-based service delivery models to reduce disability and dependency and admission to long term care, and improve quality of life.

**Methods:**

A pragmatic three parallel group randomised controlled trial involving people with Parkinson's Disease and live-in carers (family friends or paid carers), and comparing: management by a specialist multidisciplinary team for six weeks, according to a care plan agreed between the professionals and the patient and carer (Group A); multidisciplinary team management and additional support for four months from a trained care assistant (Group B); usual care, no coordinated team care planning or ongoing support (Group C). Follow up will be for six months to determine the impact and relative cost-effectiveness of the two interventions, compared to usual care. The primary outcomes are disability (patients) and strain (carers). Secondary outcomes include patient mobility, falls, speech, pain, self efficacy, health and social care use; carer general health; patient and carer social functioning, psychological wellbeing, health related quality of life. Semi structured interviews will be undertaken with providers (team members, care assistants), service commissioners, and patients and carers in groups A and B, to gain feedback about the acceptability of the interventions. A cost - effectiveness evaluation is embedded in the trial.

**Discussion:**

The trial investigates components of recent national policy recommendations for people with long term conditions, and Parkinson's Disease in particular, and will provide guidance to inform local service planning and commissioning.

**Trial registration:**

ISRCTN: ISRCTN44577970

## Background

Parkinson's disease is a degenerative neurological condition that affects mainly older people, but also significant numbers with young onset [1]. Although designated a movement disorder, it additionally inflicts a range of distressing non motor symptoms. As the disease progresses, people with Parkinson's become increasingly dependent and a considerable burden is carried by family carers. The mainstay of management is a pharmacological regimen which gradually becomes less effective, and more complicated. This is supported by rehabilitative therapies, assistive technologies and occasionally surgery. A multidisciplinary team (MDT) approach to rehabilitation is accepted best-practice [2-4], but has not been widely researched [4-7]. Whilst a self management programme has been found to have positive effects on health-related quality of life at six months [8], other community-based studies have only measured immediate outcomes [9,10], or found no longer term benefits [11]. A need has been expressed for studies that identify cost-effective service delivery models that reduce disability and dependency and prevent admission to long term care [3-7].

The SPIRiTT study (Specialist Parkinson's Integrated Rehabilitation Team Trial) builds on the findings of a previous multidisciplinary rehabilitation programme, coordinated by a Parkinson's Nurse Specialist (PNS) in a day hospital setting [12]. This intervention resulted in significant immediate gains for patients in mobility, independence, wellbeing and health-related quality of life [10], but, in the absence of continuing input, these benefits had largely dissipated four months after the intervention ended [12]. Moreover, the accompanying economic evaluation showed that day hospital treatment incurred facility overhead costs and involved the use of expensive hospital transport for patients with more advanced disease who were unable to make their own arrangements [13]. SPIRiTT specifically addresses the issue of patient transport raised by the day hospital model by delivering rehabilitation to people in their own homes. Moreover, it evaluates whether the fading of benefit when specialist input is withdrawn, (a common feature of time limited rehabilitation interventions [5]), can be avoided in a cost - effective way by providing continuing support from specially trained care assistants. Participants in SPIRiTT will receive an equivalent package of specialist rehabilitation to that used in the day hospital study so that valid comparisons can be drawn between the models of domiciliary and day hospital provision.

The SPIRiTT model of service delivery is grounded in the recommendations of several recent policy documents of the English National Health Service (NHS). These promote: the integration of health and social care services [14], provision of services closer to patients' homes [14], coordination of care for particular patient groups by specialist disease-specific nurses [15], supported self management [15], and personalised care planning, rehabilitation and carer support in order to reduce costly unplanned hospital admissions [16]. Moreover, guidelines from the National Institute for Health and Clinical Excellence (NICE) for the management of Parkinson's disease [3] recommend regular patient review, comprehensive care plans, a central role for PNS and regular access to physiotherapy, occupational therapy and speech and language therapy.

Many people with Parkinson's do not currently see individual therapists and even fewer receive coordinated MDT input [17,18]. SPIRiTT will investigate the cost-effectiveness of implementing a proactive approach to Parkinson's management, in line with recent recommendations. Other research has shown routine assessment and support for older people living in the community with a variety of conditions can have positive effects on mortality and admission to long term care [19]. Evaluations have been conducted in a range of countries, including the United States [20-23], Canada [24], Australia [25,26], Denmark [27,28], Italy [29] and Switzerland [30], but overall evidence on outcomes (such as physical functioning and health-related quality of life), service use and costs is inconsistent [26,31]. The findings from SPIRiTT will extend the current evidence base through a focus on outcomes for people with Parkinson's.

Capacity constraints in the form of high nurse caseloads and shortages of therapists were identified by NICE as barriers to the delivery of their guidance for management of Parkinson's disease [3], and these have been confirmed by a recent survey of PNS [32]. Whilst NICE recommends a caseload of 300 patients, over half of PNS have lists in excess of 500, with adverse effects on the amount of routine support they can provide to patients. In common with other advanced practice nurses in the community, PNS report undertaking a variety of tasks (some of which do not require advanced skills), and that time pressures create a need to risk stratify patients, and a focus on 'crisis' management, rather than ongoing advice and support [33,34]. Use of care assistants, trained in the special features and management of Parkinson's, working with PNS and multidisciplinary teams of healthcare professionals in the community on assigned tasks appropriate to their skill level and knowledge, is one way in which resources for delivering care and support to people with Parkinson's can be increased.

Competency-based training enables non registered staff to properly complement the activities of professionals [14,35], and professionals to appropriately meet supervision, delegation and accountability challenges [36]. Trained care assistants have been shown to be effective at underpinning professional working and to have a positive impact on nurse's ability to provide high quality care, their work experiences, and the cost-effectiveness of service delivery [37]. The use of trained assistants is consistent with NHS policy for the health and social care workforce which advocates the integration of non registered health and social care workers with enhanced roles in MDTs, to implement and deliver therapy and monitor and support patients [16,38], as a means of increasing the flexibility, efficiency and responsiveness of services [39,40].

The **aims **of the SPIRiTT study are to evaluate two models of specialist MDT rehabilitation for people with Parkinson's in the community, to add to the existing evidence base, to inform future service development and commissioning, and ultimately to improve the quality of care and outcomes for patients and family carers. The specialist intervention is based on a rehabilitation service that works with the patient and family to resolve problems, through a process of goal setting, care planning, intervention and evaluation, to achieve outcomes that maximize functioning and social participation with minimum distress to patient or family carer [41]. The research will explore not just the multidisciplinary professional input, but also budgetary and management arrangements, and barriers and facilitators to cross sector working, that may impact on future implementation of the model.

The specific **objectives **are to:

1. Implement a specialist neurological rehabilitation service for people with Parkinson's and their family carers, delivered in their own homes, comprising MDT assessment, care planning and treatment (following the protocol previously evaluated in a day hospital setting);

2. Provide ongoing support from specially trained care assistants to half (randomly selected) of those receiving the specialist rehabilitation;

3. Evaluate the clinical effectiveness of the specialist rehabilitation service, and the value added by ongoing support from trained care assistants embedded in the MDT, compared to usual care, (which is largely non specialist and non team based), across a range of patient and carer outcomes;

4. Assess the costs of the specialist rehabilitation intervention, and of the ongoing care assistant support, and calculate relative cost-effectiveness, including consideration of savings from service use offsets;

5. Investigate the acceptability of the new service delivery models (specialist domiciliary rehabilitation with and without ongoing support from trained care assistants) from the perspectives of all stakeholders including commissioners, MDT members, care assistants, service managers, patients and family carers;

6. Deliver guidance for commissioners, providers and policy makers about the acceptability, clinical and cost-effectiveness of different models of specialist neurological rehabilitation.

The **hypotheses **are that:

1. A package of domiciliary multidisciplinary specialist rehabilitation will benefit:

a. People with Parkinson's in terms of maintaining mobility and independence (primary outcome for patients), and improving wellbeing and health-related quality of life,

b. Family carers in terms of reduced strain (primary outcome for carers), and improved health-related quality of life, and

c. Society through reduced use of other health and social care services, including hospitalisations and admissions to long term care.

2. The addition of four months of ongoing support from trained care assistants will help to maintain the benefits of the specialist team rehabilitation, and avoid the fading of effects that typically accompanies the withdrawal of input.

3. The intervention will be acceptable to major stakeholders, and that barriers and facilitators to wider implementation will be identified.

## Design/Methods

### Design

**Figure 1:**
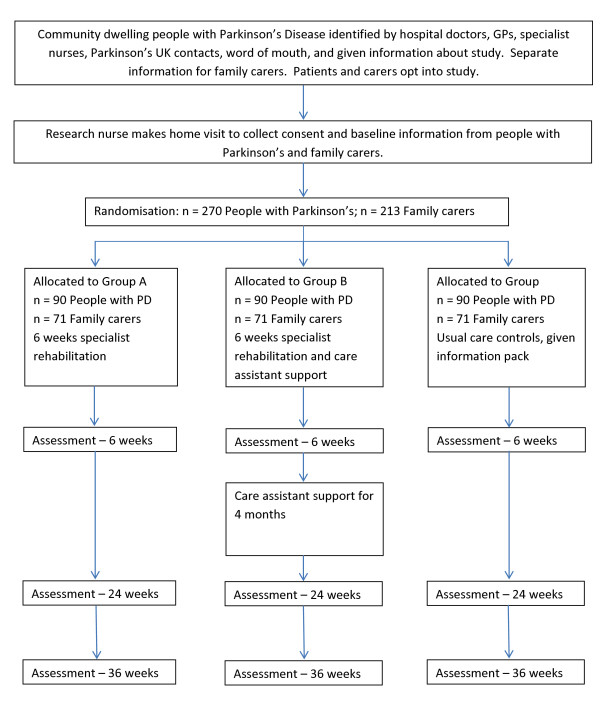
**CONSORT Flow Chart for SPIRiTT (Specialist Parkinson's Rehabilitation Team Trial)**.

A pragmatic three parallel group randomised controlled trial. People with Parkinson's in group A will be assessed and managed by a specialist MDT according to a care plan that is agreed amongst the professionals and with the patient and carer. Group B will additionally receive ongoing support for four months from a trained care assistant. Group C will receive normal care (no coordinated MDT care planning or ongoing support). Follow up will be for six months to determine the impact and relative cost-effectiveness of the two interventions. Qualitative interviews will be undertaken with providers (MDT members, care assistants), service commissioners, and patients and carers in groups A and B, to gain feedback about the acceptability of the interventions. The CONSORT diagram [42] summarises the design (Figure 1).

### Setting

Contiguous communities around three district general hospitals (DGHs) in the county of Surrey, England. The study area contains urban, suburban and rural localities, and a broad mix of socio-economic and ethnic groups.

### Participants

People with Parkinson's (all stages of the disease) and live-in carers (where applicable).

### Recruitment

People with Parkinson's disease will be identified by a variety of means including: hospital clinics; general practitioners (GPs); Parkinson's UK contacts; PNS, community-based therapists; word of mouth. Research nurses from the Primary Care Research Network (PCRN) and the Dementia's and Neurodegenerative Diseases Research Network (DeNDRoN) will assist with the identification of people with Parkinson's through general practices and specialist Parkinson's hospital clinics respectively. Any interested person with Parkinson's will be given an information leaflet. Separate information will be provided for live-in carers (family, friends, paid carers living in the same household), where appropriate. People with Parkinson's and live-in carers will volunteer separately to take part in the study by contacting the research team, who will undertake an initial eligibility screen. This screening will include asking the patient to confirm that a doctor has told them that they have Parkinson's disease. Volunteers who meet the inclusion criteria (see below) will be sent full information about the trial, and a consent form, and an appointment will be made for a research nurse to make a home visit to collect baseline information. If a live-in carer does not want to take part in the research, the person with Parkinson's may still join the trial. However, carers will not be accepted if the person with Parkinson's does not want to participate.

### Inclusion criteria

People with Parkinson's (any stage of the disease) will be included if they: are 18 years of age or over; have a clinical diagnosis of Parkinson's disease; are living in the community (own home or minimally sheltered accommodation) with their own living areas; live in the county of Surrey (for reasons associated with the funding of the intervention); are able to read and write English in order to complete the self report questionnaires; express a commitment to participate over the duration of the study and provide written consent.

Live-in carers will be included if they: are 18 years of age, or over; are able to read and write English in order to complete the self report questionnaires; express a commitment to participate over the duration of the study and provide written consent.

### Consent and baseline data collection

Volunteers will be entered into the trial in blocks of thirty (geographically defined in order to reduce travel time and costs to participants' homes for the collection of the research data and delivery of the intervention). Research nurses will make a home visit to collect written consent(s) and record background information (age, gender, time since diagnosis, disease stage, co-morbidities, housing, caring arrangements, income and benefits, social support - Lubben Social Network Scale [43], cognitive function - mini mental state examination [44]). A baseline assessment will also be conducted using the outcome measures selected for the trial (see Table below). The intervention for each block of participants will start within two weeks of completion of baseline data collection.

### Exclusion criteria

Exclusion criteria will be checked by the research nurse at the baseline assessment. People with Parkinson's will be excluded if they: score at the most favourable end of all outcome scales at baseline, because the trial cannot demonstrate an improvement in these patients, and, in six months, has little likelihood of demonstrating reduction in any expected decline; score less than 24 on the mini mental state examination at baseline assessment [44], to ensure those recruited can follow instructions associated with the rehabilitation intervention; are already having a multidisciplinary package of care, or have had such care in the last six months; are taking part in another rehabilitation-based research study, or have taken part in such research in the last six months.

### Registration and randomisation

After consent and baseline data have been collected, eligible volunteers will be given a unique registration number by the project administrator and randomised to either: Group A - specialist rehabilitation, Group B - specialist rehabilitation and ongoing care assistant support, or Group C - usual care, control group. A separate randomisation sequence will be prepared by the study statistician prior to the commencement of the study for patients without live-in carers and for patients with live-in carers. In each instance, blocked randomisation will be used to formulate the sequence involving the three comparison groups. A non-computerised random number generator will create random permuted blocks of size three with an equal allocation ratio (with any of the six different group sequences being equally possible). The project administrator will inform: all participants of the group to which they have been randomised; the MDT of participants randomised to either of the treatment arms; the GPs of all participants about their involvement in the trial and the group to which they have been randomised. Since the project administrator will not be involved in the process of consenting volunteers into the study, and randomisation of eligible participants will be conducted in chronological order of study entry, no attempt will be made to conceal from the project administrator the next group to which the next patient would be allocated. However, only the project administrator and the study statistician will have access to each randomisation sequence.

### Interventions

#### Specialist rehabilitation intervention (Groups A and B)

A multidisciplinary team comprising two each of PNS, physiotherapist, occupational therapist, and speech and language therapist will be assembled from local professionals. They will work one day per week for the trial, and in other duties for the rest of the time. Team members will visit the homes of participants to deliver a specialist rehabilitation package, tailored to individual needs. In order to make the outcome from the trial comparable to that of the previous study set in a day hospital [10-12], a similar programme of specialist rehabilitation will be provided comprising an initial assessment, and the formation of an agreed care plan reflecting the needs, wishes and expectations of the person with Parkinson's and carers. A group education component in the day hospital trial cannot be replicated in the domiciliary setting. As a substitute, the MDT will provide participants with a folder containing 13 fact sheets produced by Parkinson's UK and the research team, covering various aspects of living with Parkinson's including: medications, physiotherapy exercises, foot care, diet and nutrition, speech and language, relaxation techniques, sleep and fatigue, continence and bowel care, rights and benefits and advice for carers.

The rehabilitation intervention will be coordinated by the PNS, and will involve specialist input from each professional, over a period of six weeks. The team will meet face-to-face four times in each six week cycle to discuss patient plans and progress, and communicate by email and telephone at other times. Two hospital consultants (neurologist and geriatrician), both with a special interest in movement disorders, can be called upon by the MDT for medication changes or advice. Referrals to a range of other professionals will be made as required, including community psychologist, social care manager, continence adviser, dietician and pharmacist. Overall three days of professional time (including travel and meeting time) is allowed for each person with Parkinson's in the trial, but some people may need more and others less than this. This input is largely equivalent to the 9-12 hours of individualised nursing and therapy input, and flexible access to other professionals, in the previous day hospital trial.

#### Ongoing support (Group B)

In addition to the programme of specialist MDT rehabilitation, participants randomised to Group B will receive ongoing support for four months from a care assistant trained in Parkinson's, starting at the end of the six week MDT intervention. The care assistants will be part of the MDT and will work under the supervision of the PNS. About one hour per week per patient is allowed for ongoing support, and contact will to be through a mix of home visits and telephone, through which the care assistant will monitor progress in implementation of the agreed care plan and report back to the MDT. If required, MDT members may continue to provide input. Care assistants will be recruited to the project from local health and social care employers and trained using the material developed by the research team [45], and through shadowing MDT members.

#### Usual Care/Control (Group C)

Participants in the control group will continue to receive care as usual (no coordinated MDT care planning or ongoing support). When informed of their group allocation, they will be sent locally relevant and generic information about Parkinson's disease. This is a small enhancement on the service they are likely to be receiving. In order to measure the impact of the interventions, this information will also be given to the participants in Groups A and B. At the end of the trial, people in the control group will be offered an assessment by the MDT, with advice, and the fact sheets provided to groups A and B, but additional services cannot be guaranteed.

Cross contamination between groups will be minimal because patients will be individually recruited to the study, and receive treatments tailored to their specific needs. Moreover, the intervention and research assessments will take place in participants' homes which will be geographically dispersed over the catchment areas of three large district general hospitals.

### Outcome measures

Participants in all groups will be assessed in their homes by a research nurse, according to the schedule used in the day hospital trial, at weeks 6 (after the rehabilitation intervention), 24 (four months after the end rehabilitation, coinciding with the end ongoing support for Group B), and 36 (for final follow up). Validated instruments which reflect the needs of participants (e.g. functional outcomes, quality of life) and service commissioners (e.g. service use), and which have been found sensitive in previous rehabilitation studies undertaken by the research team [10-13] have been selected to measure outcomes (Table 1).

**Table 1:** Trial outcome measures and instruments: baseline, 6, 24 and 36 weeks

Participant	Outcome measure	Instrument
Person with Parkinson's	Parkinson's disability/general activities	Self Assessment Parkinson's Disease Disability Scale [46] (PRIMARY OUTCOME)
	
	Parkinson's specific	Parkinson's Disease Questionnaire [47]
		
		Non Motor Symptom Questionnaire [48]
	
	Mobility	Posture and gait items from the Unified Parkinson's Disease Rating Scale [49]
		
		Timed up and go [50]
	
	Falls	Self report
	
	Speech	Single speech item from the Unified Parkinson's Disease Rating Scale [49]
		
		Abridged Emerson and Enderby Screening Assessment Rating Scale [51]
		
		Frenchay Dysarthria Summary [52]
		
		Speech self report questionnaire [10-12]
	
	Pain	Visual analogue scale [53]
	
	Generic health related quality of life/QALYs	Euro Qol 5D, with utility index for calculation of quality adjusted life years (QALYs) [54]
	
	Self efficacy	Self efficacy scale [55]
	
	Health and social care utilisation	Customised proforma and patient records

Live-in carer	Carer strain	Modified caregiver strain index [56] (PRIMARY OUTCOME)
	
	General health	General health questionnaire - 12 [57]

Both	Activities of daily living	Barthel ADL index [58]
	
	Social activities	Frenchay Activities index [59]
	
	Psychological wellbeing	Hospital Anxiety and Depression Scale [60]
		
		Yale single item depression screening tool [61]
	
	Generic health related quality of life	Short form - 36 Health Survey [62]

The instrument battery was developed and piloted in collaboration with patient and carer representatives. It takes about one hour to complete, and was not been found too onerous. Research nurses will assist patients with completion of questionnaires. Carers will be asked to self complete the questionnaires that apply to them. All questionnaires will be checked for completeness before the research nurse leaves the participant's home. Data will be entered by the research nurse shortly after collection, and any queries referred back to the participant by telephone. A further check on data (completeness and entry) will be undertaken by the research manager when forms are returned to the office by the research nurse, and missing items or other issues will be resolved. Patient and carer outcomes will be analysed separately.

### Blinding

The research administrator will not disclose group allocation to the research nurses who undertake assessments, and participants will be asked not to discuss aspects of the trial and treatments with them. However, it is recognised that blinding of the research nurses may be compromised in trials of this nature. As a check on blinding, research nurses will be asked to guess participants' groups at the end of the trial. For data analysis, the group identifiers will not be disclosed to the statistician.

### Acceptability of the intervention

Semi structured interview schedules will be designed and used to gather feedback on the acceptability of the interventions from group A and B participants during the 24 week research assessment. This part of the study will capture the patient and carer voice and experience of the rehabilitation interventions relative to perceived needs and priorities. In addition, service providers, MDT members, care assistants and a selection of commissioners will be asked for their views about the interventions, to identify strengths and weaknesses, barriers and facilitators to the wider use of the interventions.

### Sample size calculations

Patient sample size calculations were based on detecting clinically meaningful differences in the primary patient outcome measure. Carer sample sizes reflect the findings of our previous work that suggest that 79% of people with Parkinson's have a carer [10].

We will recruit 270 people with Parkinson's over a 12 month period across the three areas, 90 of whom will be randomly allocated to each of the three groups. This calculation is based on the numbers of people with Parkinson's needed to detect differences between groups in patient primary outcome measure score.

Assuming a similar level of variation in Self Assessment Parkinson's Disease Disability Scale [46] to the day hospital trial [11], in order to detect a difference in the disability score of 1.25 (assuming SD = 2.5, size = 5%, power = 80% and a 2 sided test), 64 subjects with Parkinson's disease are needed in each of the three groups.

Assuming a similar level of variation in Carer Strain Index [56] to the day hospital trial [11], in order to detect a difference in the Carer Strain Index of 0.535 (assuming SD = 1.07, size = 5%, power = 80% and a 2 sided test), 64 carers are needed in each of the three groups. In the previous study, 79% of community dwelling people with Parkinson's had a live-in carer [10]. Thus, if we have 64 carers per group, this will necessitate 246 [(64*3)/0.79 = 243.04] Parkinson's subjects, i.e 82 per group.

In the previous day hospital trial the loss to follow-up/non completion/missing data rate between recruitment and six month assessments was 26%. However, in this trial, participants attended the day hospital for treatment (six visits) and research assessments (four visits), and difficulties with transport and inter-current illness accounted for missing data and drop-out. We expect less attrition in the SPIRiTT trial because participants will receive both treatment and the research assessments in their own homes, at times convenient to them.

If we allow for 10% loss to follow up/non completion/missing data rates for people with Parkinson's, then 243.04/.90 = 270.03 patients are required = 90 per group. With 90 patients per group, we expect to recruit 71 carers per group. These group sizes will also ensure that the samples for patients and carers will both remain above the critical values of 64 if there were a loss of 5% of carers (and associated patients) and an independent loss of 5% of people with Parkinson's. Intention to treat analysis will be used.

### Withdrawals

Participants may withdraw from the study due to illness or personal reasons. They will be made aware (via the information sheet and consent form) that withdrawal from the study will not affect their future care, and that data collected to date will still be used in the final analysis. Volunteers who are not randomised because they fail eligibility criteria at baseline will be replaced, but participants who withdraw from the trial for any reason will not be replaced.

### Statistical methods

Data will be entered into an SPSS (latest version) computer data-base using the registration number to identify the participant. All paper data will be stored in a secure locked cabinet, and all computer data will be stored on a secure server.

Baseline data will be analysed to describe the characteristics of the participants and to check for significant imbalance between the three groups. All outcomes will be analysed at each follow up assessment point (six, 24 and 36 weeks), and specific *a priori *hypotheses will be tested reflecting the outcomes that we expect to identify at the different stages. In each case a two sided test will be used. It is expected that t tests will be conducted to compare groups, unless any obvious deviations from t test assumptions are found in the outcomes, in which case the non - parametric Mann Whitney U test will be used.

In order to identify short term benefits, participants in the specialist rehabilitation groups (A and B) will be compared with participants receiving information pack only (group C) at six weeks. The null hypotheses will be tested, that there are no differences between the groups, with respect to change in patient disability, mobility and psychological wellbeing, and in carer strain. These hypotheses will be tested using each participant's change score from baseline (week 0) to week 6.

In order to identify medium term benefits, comparisons will be made at 24 weeks between pairs of treatment groups (Group A vs. Group B; Group A vs. Group C; Group B vs. Group C). The null hypotheses will be tested, that there are no differences between the groups with respect to change in patient disability, mobility, falls, speech, activities of daily living, social activities, psychological wellbeing, health related quality of life and use of health and social care services, and in carer strain, social activities and health related quality of life. These hypotheses will be tested using each participant's change score from baseline (week 0) to week 24.

In order to show loss or maintenance of benefit resulting from specialist rehabilitation, Group A (no ongoing support weeks 7 and 24) will be compared with Group B (ongoing support weeks 7 to 24) using the hypotheses in the medium term analysis and each participant's change score from week 6 to week 24.

In order to identify longer term benefits, comparisons will be made at 36 weeks between pairs of treatment groups (Group A vs. Group B; Group A vs. Group C; Group B vs. Group C). The null hypotheses will be tested, that there are no differences between the groups with respect to change in patient disability, mobility, falls, speech, activities of daily living, social activities, psychological wellbeing, health related quality of life and use of health and social care services, and in carer strain, social activities and health related quality of life. These hypotheses will be tested using each participant's change score from baseline (week 0) to week 36.

An additional exploratory analysis will be performed using each participants change score for the above outcomes between week 24 and week 36. This is the follow-up period, and this analysis will provide evidence of trends in each group, and difference between groups, after all interventions cease in week 24.

It is recognised that multiple statistical tests are being undertaken, but no adjustments will be needed for multiple testing because *a priori *hypotheses have been stipulated, and the selective reporting of significant results will be avoided. Any additional exploratory (hypothesis generating) data analyses will make allowance for multiple testing in the usual way (Bonferroni).

### Missing data

Stringent attempts will be made to minimise missing data through checking of questionnaires as they are completed, and returning to participants to retrieve missing items. Participants with missing outcome data at a specific time point will be excluded from analysis at that stage. Multiple imputation will be used to estimate missing outcome scores in repeated measures analysis.

### Economic evaluation

The resources used in the delivery of the interventions will be collected at the patient level from records kept by the MDT (Groups A and B), and the care assistants (Group B only). These data will include details of patient contact (number and duration of visits or telephone calls), travel time and distance for home visits, materials, consumables or equipment used, time spent liaising with other professionals (e.g. consultant, GP), and referrals made. Administrative and meeting time of the MDT will be averaged across participants in recruitment blocks of thirty. Monetary costs will be assigned to all resources used to ascertain a cost for each participant, and group means and variation calculated and compared. Unit costs of resources will be based on data from local financial managers, validated national sources [63], and market prices, as appropriate, and multiplied by the number of units used.

The use of health and social care services (GP, community, outpatient, A&E, hospital inpatient) will be collected by self report at each assessment point by recall for the previous period (three months prior to baseline). Responses will be verified as far as possible (within the resources of the trial) from local records. Unit costs of services will be obtained from local and national sources [63] and multiplied by the number of units used. Costs of service utilisation in the intervention groups (A and B) will be compared with each other and with the usual care group (C) to assess the extent to which the interventions may be offset by savings elsewhere in the health and social care system.

The economic analysis will evaluate the costs of the intervention in relation to all patient and carer consequences/outcomes [64]. In addition, a cost-effectiveness analysis will be conducted, using standard techniques of economic appraisal [65]. Mean differences between groups (Group A (specialist rehabilitation only) vs. group C (normal care controls); Group B (rehabilitation with ongoing support) vs. Group A) in the primary outcome measures (disability score for patients, carer strain) and in QALYs (patients only) adjusted for baseline values in an analysis of covariance, will be used to measure effectiveness. If statistically significant differences between groups are found, incremental cost-effectiveness ratios will be calculated to show the extra cost incurred per unit of therapeutic gain/QALY gain. Secondary cost-effectiveness analyses will be conducted using other outcomes as measures of effectiveness.

The impact of uncertainties in the estimation of costs and outcome variables will be explored using one way and probabilistic sensitivity analysis. Bootstrap methods will be used to represent uncertainty of estimates, either for constructing confidence intervals or probability curves. Sensitivity analysis will also investigate the cost-effectiveness of the interventions for people with different levels of disability at baseline.

### Risks and adverse events

An adverse event (AE) is any unfavourable or unintended sign, symptom, syndrome or illness that develops or worsens during the period of the trial. A serious adverse event (SAE) is any adverse event which is life threatening, or results in hospitalisation, disability or death. AE and SAE will be identified by research or intervention team members, or reported by participants. They will be recorded, assessed for seriousness, expectedness and causality by clinical members of the research team and monitored. Any SAE deemed to be directly related to, or suspected to be related to the intervention and unexpected will be reported to the study Steering Group and the Ethics Committee.

Risks to participants from the trial are considered small, and no higher than those of usual care. The aspects of the MDT intervention delivered by the individual therapists are standard practice and aim to improve self awareness and management. Home visits will include assessment of home safety and aids and adaptations required which may require in improvements in safety for participants. However, it is possible that encouragement to exercise more could result in falls that might not otherwise have occurred. The MDT members experienced professionals and will emphasise safety issues to participants. The care assistants will be fully trained, and will work under the instruction of team professionals, in the support of patients and carers and the implementation of the agreed care plan. Other potential harms to the experimental groups include depression if raised expectations are not met, distress when additional input is stopped, and loss of support from family and friends if the additional care is perceived to reduce the need for informal support.

### Management and governance

The research team comprises a chief investigator (HG) with overall responsibility for the conduct of the research, two clinical principal investigators (BC, PT), a full time research manager (ST), a qualitative researcher (KB), a statistician (PW), research advisor (DW), and a co-ordinator of public and patient involvement (JK). All aspects of data collection and management are under the supervision of the research manager. The research team is supported in the delivery of the trial by an External Steering Committee, which meets twice per year to review progress and ensure timely completion of milestones. Membership of the EAG includes clinical experts, experienced researchers, a statistician, representatives from Parkinson's UK and the European Parkinson's Disease Association, local service providers and commissioners, and people with Parkinson's and carers. A separate PPI (Patient and Public Involvement) group, co-ordinated by a Parkinson's Specialist Nurse, helps the research team with the development of study documents and processes, and was involved in the planning stage of the project.

### Ethical and organisational review

Ethical approval has been granted to the Surrey Research Ethics Committee (application number 10/H1109/1). National Health Service Research and Development approval has been granted by four Participant Identification Centres.

## Discussion

The SPIRiTT trial is a community based MDT rehabilitation intervention for people with Parkinson's disease and their live-in carers. The intervention is delivered to people in their own homes, so that the costs and effectiveness can be compared with a similar intervention delivered to the same patient group in a day hospital setting. Findings will be compared with another community-based RCT, conducted in England recently that found no significant difference in outcomes or costs of patients receiving rehabilitation in a day hospital rather than in their own homes [66].

We anticipate several problems in the conduct of the trial and will put processes in place to avert or minimise their effect. First, retention of participants, particularly in the control group, may be difficult so we will seek to retain their interest by providing regular newsletters reporting trial progress. Patients randomised to the control group will be offered an MDT assessment and advice on completion of the trial follow up. Second, disruptions to the delivery of the MDT intervention may arise due to bad weather preventing travel, or staff sickness. We expect such interruptions will be minimal and consistent with a pragmatic approach to research on service delivery, and that cover will be available within the team for short term holiday or sickness leave. Third, lack of local NHS resources, due to financial exigencies, could impede the delivery of the intervention according to protocol to the numbers of patients and carers required by the sample size calculations, but we will liaise with local commissioners and providers to try and ensure their support. Fourth, recruiting MDT members and care assistants may be problematical, due to difficulties arranging temporary secondments from local providers, and local labour market conditions. We will address any staffing issues by advertising widely and offering favourable contracts for suitably qualified applicants. Lastly we may encounter difficulties in persuading live-in carers to take part in the research assessments, and we will seek to overcome this by explaining why their participation is important to the findings.

The trial investigates important issues in recent national policy recommendations for people with long term conditions, and Parkinson's disease in particular, and will provide guidance to inform local service planning and commissioning. The population of the study area is diverse and findings are more widely generalisable.

### Trial status

Ongoing

## List of abbreviations

AE: Adverse event; SAE: Serious adverse event; DeNDRoN: Dementia's and Neurodegenerative Diseases Research Network; DGH: District General Hospital; GP: General Practitioner; MDT: Multi disciplinary team; NHS: National Health Service; NICE: National Institute of Health and Clinical Excellence; PCRN: Primary Care Research Network; PNS: Parkinson's Nurse Specialist; QALY: Quality adjusted life years; SPIRiTT: Specialist Parkinson's Integrated Rehabilitation Team Trial

## Competing interests

The authors declare that they have no competing interests.

## Authors' contributions

HG, JK, PT, DW, PW, KB, BC developed the research protocol for funding, and together with ST wrote and refined the trial protocol. All authors have read and approved the final manuscript.
